# Loss of Angiotensin II Type 2 Receptor Improves Blood Pressure in Elastin Insufficiency

**DOI:** 10.3389/fcvm.2021.782138

**Published:** 2021-11-01

**Authors:** Michelle Lin, Robyn A. Roth, Beth A. Kozel, Robert P. Mecham, Carmen M. Halabi

**Affiliations:** ^1^Division of Nephrology, Department of Pediatrics, Washington University School of Medicine, Saint Louis, MO, United States; ^2^Department of Cell Biology and Physiology, Washington University School of Medicine, Saint Louis, MO, United States; ^3^National Heart, Lung and Blood Institute, National Institutes of Health, Bethesda, MD, United States

**Keywords:** angiotensin II type 2 receptor, elastin insufficiency, hypertension, vascular stiffness, vascular biology

## Abstract

There is ample evidence supporting a role for angiotensin II type 2 receptor (AT_2_R) in counterbalancing the effects of angiotensin II (ang II) through the angiotensin II type 1 receptor by promoting vasodilation and having anti-inflammatory effects. Elastin insufficiency in both humans and mice results in large artery stiffness and systolic hypertension. Unexpectedly, mesenteric arteries from elastin insufficient (*Eln*^+/−^) mice were shown to have significant vasoconstriction to AT_2_R agonism *in vitro* suggesting that AT_2_R may have vasoconstrictor effects in elastin insufficiency. Given the potential promise for the use of AT_2_R agonists clinically, the goal of this study was to determine whether AT_2_R has vasoconstrictive effects in elastin insufficiency *in vivo*. To avoid off-target effects of agonists and antagonists, mice lacking AT_2_R (*Agtr2*^−/*Y*^) were bred to *Eln*^+/−^ mice and cardiovascular parameters were assessed in wild-type (WT), *Agtr2*^−/*Y*^, *Eln*^+/−^, and *Agtr2*^−/*Y*^*;Eln*^+/−^ littermates. As previously published, *Agtr2*^−/*Y*^ mice were normotensive at baseline and had no large artery stiffness, while *Eln*^+/−^ mice exhibited systolic hypertension and large artery stiffness. Loss of AT_2_R in *Eln*^+/−^ mice did not affect large artery stiffness or arterial structure but resulted in significant reduction of both systolic and diastolic blood pressure. These data support a potential vasocontractile role for AT_2_R in elastin insufficiency. Careful consideration and investigation are necessary to determine the patient population that might benefit from the use of AT_2_R agonists.

## Introduction

Elastin (ELN), the main component of elastic fibers, is responsible for conduit arteries' elastic recoil. This recoil is necessary to dampen the pulsatile flow of ventricular ejection at the level of the ascending aorta and transform it into continuous flow at the level of arterioles or small resistance arteries. Elastic fibers are organized into fenestrated concentric sheets or lamellae in blood vessels. Decreased elasticity of large arteries with aging is attributed to fragmentation and thinning of these lamellae and results in increased pulse wave velocity leading to a greater augmentation of the central aortic systolic and pulse pressures (1, 2). Similarly, genetic reduction of elastin through deletion of a single copy of the gene *ELN* (supravalvular aortic stenosis—SVAS, OMIM #185500) or deletion of *ELN* as part of a 25–27 coding gene microdeletion of chromosome 7 (Williams syndrome, OMIM #194050) leads to increased pulse wave velocity and hypertension (3–5).

Similar to humans with SVAS and Williams syndrome, mice hemizygous for the elastin gene (*Eln*^+/−^) develop large artery stiffness and systolic hypertension (6, 7). Interestingly, the increased large artery stiffness in *Eln*^+/−^ mice precedes the appearance of hypertension (8) and is not affected by commonly used anti-hypertensives (9). Increased large artery stiffness and central systolic and pulse pressures often lead to structural and functional changes in small resistance arteries that further exacerbate hypertension and a vicious cycle ensues (10). This appears to be the case in elastin insufficiency as recent studies showed altered resistance vessel reactivity that is vascular bed-specific (11–13). Mesenteric arteries (MAs) and middle cerebral arteries (MCAs), but not gastrocnemius feed arteries (GFAs), were found to have impaired endothelial-dependent dilation to acetylcholine due to decreased nitric oxide availability resulting from increased oxidative stress (11, 12, 14). Furthermore, MAs and MCAs, but not GFAs, had an increased contractile response to angiotensin II (ang II) (11, 13). Interestingly, the hypercontractile response of mesenteric arteries to ang II was mediated, at least in part, by the angiotensin II type 2 receptor (AT_2_R) as blockade of AT_2_R with the antagonist PD123319 decreased the contractile response of MAs to ang II while its activation with novokinin resulted in vasoconstriction (11).

Given the multitude of evidence suggesting a vasodilatory role for AT_2_R particularly in disease states and the consideration for the use of AT_2_R agonists for patients with COVID-19 and idiopathic pulmonary fibrosis among others (clinicaltrials.gov), we sought to determine the cardiovascular role of AT_2_R in elastin insufficiency *in vivo*. We bred elastin insufficient (*Eln*^+/−^) mice to AT_2_R knock-out (*Agtr2*^−/*Y*^) mice and examined cardiovascular endpoints. While loss of AT_2_R did not affect large artery structure or function, it lowered blood pressure in elastin insufficient mice, suggesting that AT_2_R plays a vasocontractile role in elastin insufficiency. This observation has significant therapeutic implications since AT_2_R agonists, which may be beneficial in some conditions such as stroke (15, 16), aneurysm formation (17, 18) and myocardial fibrosis (19), would not be appropriate in patients with elastin insufficiency.

## Materials and Methods

### Mice

*Eln*^+/−^ mice backcrossed into the 129X1/SvJ background (14) over 10 times and the genetic background confirmed by single nucleotide polymorphism genotyping were bred to *Agtr2*^−/−^ mice maintained on the FVB/n background (20). The *Agtr2*^−/−^ mice were obtained from Dr. Curt Sigmund, with permission from Dr. Victor Dzau. Tail DNA was used to genotype the mice. Genotyping for *Eln* was done as previously described (21). The following primers were used in one PCR reaction to genotype for *Agtr2*: AT2-F GTGGTCTCACTGTTTTGTTGTC, AT2-R-WT GTATTCAATGGTTCTGACATCC, and AT2-R-KO TGCAATCCATCTTGTTCAATGGC, resulting in a 374 bp product in the WT case and a 570 bp product in the knock-out case. Since *Agtr2* is on the X chromosome and littermates were used for the studies, male mice were used for the physiologic studies to reduce the number of animals needed. Mice were housed under standard conditions with free access to food and water. All surgical procedures were performed in accordance with protocols approved by the Institutional Animal Care and Use Committee of Washington University School of Medicine.

### Blood Pressure and Heart Rate Measurement

While sedation is known to lower blood pressure and heart rate, invasive blood pressure measurement provides a more accurate assessment of central arterial pressure compared to tail cuff measurement. Unfortunately, the small caliber and tortuosity of *Eln*^+/−^ carotid arteries makes blood pressure measurement via telemetry technically challenging, therefore we measured central arterial pressure invasively under sedation. The anesthetic used, isoflurane, has the least effects on the cardiovascular system among commonly used anesthetics (22). Briefly, 3–4 month-old mice were anesthetized with 2% isoflurane and maintained at 37°C using a heating pad and a rectal thermometer for monitoring. The right common carotid artery was exposed and a Millar pressure transducer (model SPR-671) was introduced and advanced to the ascending aorta. After instrumentation was complete, isoflurane anesthesia was reduced to 1.5% and systolic blood pressure, diastolic blood pressure, and heart rate were recorded using the PowerLab data acquisition system (ADInstruments). The average of a 3-min period of stable recording was reported. The data were analyzed using LabChart 8 for Mac software (ADInstruments).

### Pressure Myography

Ascending aorta and left common carotid artery of 3–4 month-old mice were excised and placed in physiologic saline solution (PSS) composed of 130 mM NaCl, 4.7 mM KCl, 1.6 mM CaCl_2_, 1.18 mM MgSO_4_-7H_2_O, 1.17 mM KH_2_PO_4_, 14.8 mM NaHCO_3_, 5.5 mM dextrose, and 0.026 mM EDTA (pH 7.4). Vessels were cleaned of surrounding fat, mounted on a pressure arteriograph (Danish Myo Technology) and maintained in PSS at 37°C. Vessels were visualized with an inverted microscope connected to a CCD camera and a computerized system, which allows continuous recording of vessel diameter. Intravascular pressure was increased from 0 to 175 mmHg by 25-mmHg increments, the vessel outer diameter was recorded at each step (12 s per step). The average of three measurements at each pressure was reported.

### Alexa-633 Hydrazide Staining

Ascending aorta were dissected and frozen in optimal cutting temperature (OCT) compound (Sakura Finetek) at −80°C. Using a cryostat, 3-μm sections were obtained and fixed in 4% paraformaldehyde for 10 min at 4°C. Sections were washed twice with 1 × PBS for 5 min each and then incubated in 1:1,000 of a 2 mM Alexa Fluor 633 hydrazide (Life Technologies) stock in 1% bovine serum albumin (BSA)/1% fish gelatin/0.05% Triton-X in 1 × PBS for 5 min at room temperature. Sections were then washed twice with 1 × PBS for 5 min each. Slides were mounted with DAPI Fluoromount-G (SouthernBiotech) and coverslipped. Images were obtained using a Zeiss Axioskop 50 microscope and QCapture Pro software (Media Cybernetics Inc.).

### Transmission Electron Microscopy

After isolation, mesenteric arteries from 3 to 4 month-old mice were fixed in 2.5% glutaraldehyde and 0.1 M sodium cacodylate at 4°C overnight. Vessels were then sent to Washington University's Center for Cellular Imaging for processing and thin sectioning. Images were taken using a JEOL JEM-1400 Plus transmission electron microscope that is equipped with an Advanced Microscopy Techniques XR111 high-speed, 4,000 × 2,000–pixel, phosphor-scintillated, 12-bit charge-coupled device (CCD) camera.

### Statistical Analysis

One-way or two-way analysis of variance with Tukey's multiple comparisons test was used to determine differences between genotypes, as indicated in each figure legend. Statistical analyses were run using Prism 9 for Mac OS X (GraphPad Software Inc.). Data are presented as means ± SD. Differences were considered statistically significant when *P* was equal to or less than 0.05.

## Results

### Loss of AT_2_R Reduces Blood Pressure in Elastin Insufficient Mice

**Figure 1:**
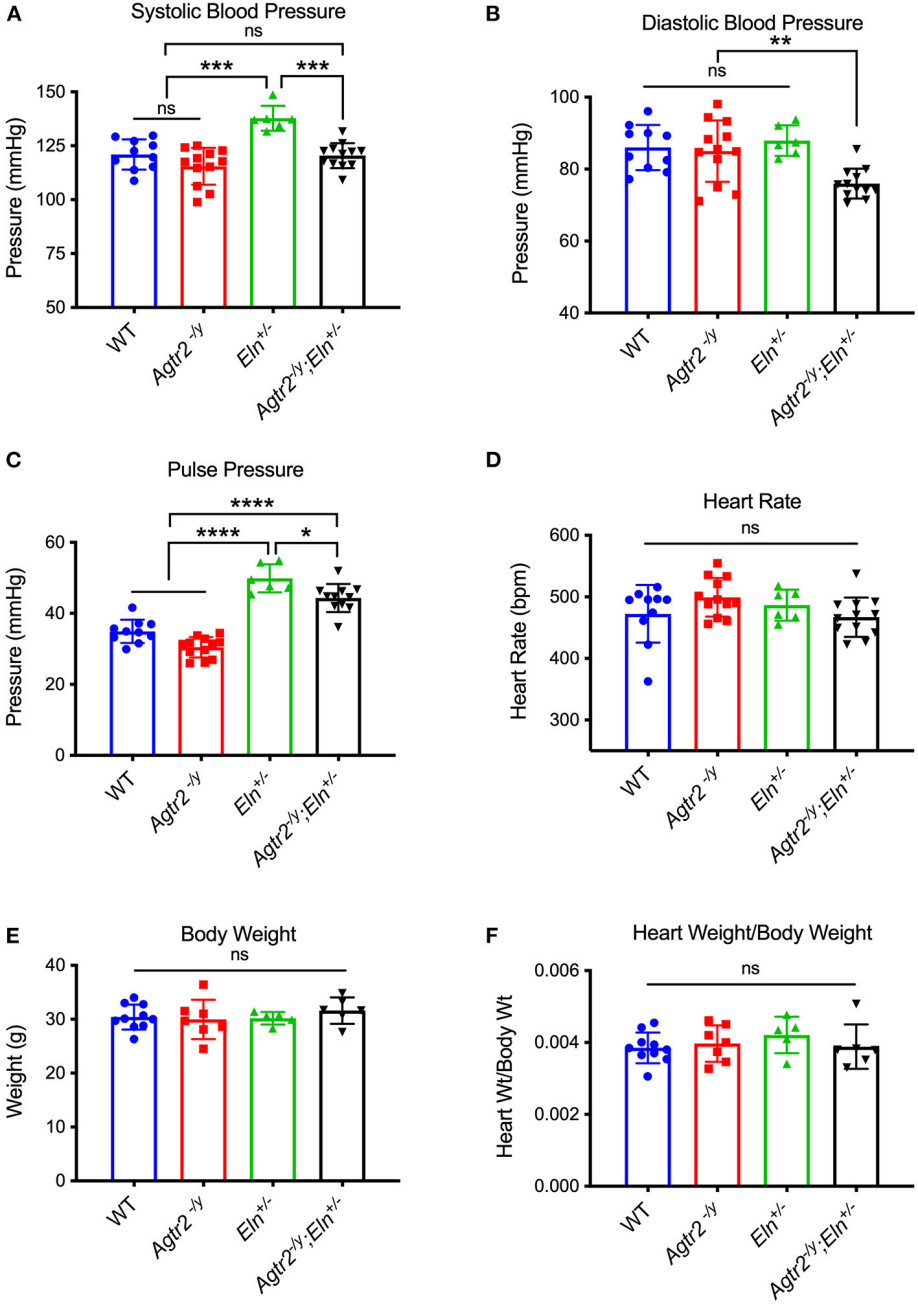
Loss of AT2R leads to a reduction in systolic and diastolic blood pressure in elastin insufficient mice. Systolic **(A)**, diastolic **(B)**, and pulse pressure **(C)** [calculated as systolic–diastolic blood pressure], heart rate **(D)**, body weight **(E)** and heart weight/body weight **(F)** of WT, *Agtr2*^−/*Y*^, *Eln*^+/−^ and *Agtr2*^−/*Y*^*;Eln*^+/−^ mice. Data are presented as mean ± standard deviation. One-way analysis of variance with Tukey's multiple comparison test was performed to compare all groups. Significant difference: *P < 0.05, **P < 0.005, ***P < 0.001, and ****P < 0.0001, between indicated groups.

To determine the role, if any, of AT_2_R in blood pressure regulation in elastin insufficiency, we bred *Agtr2*^−/*Y*^ to *Eln*^+/−^ mice. As previously reported, loss of AT_2_R did not affect blood pressure at baseline (20) and *Eln*^+/−^ mice exhibited systolic hypertension compared to wild-type (WT) littermates (6) (Figures 1A–C). Interestingly, loss of AT_2_R in elastin insufficient mice (*Agtr2*^−/*Y*^*;Eln*^+/−^) resulted in reduction of not only systolic, but also diastolic blood pressure (Figures 1A–C). Heart rate, body weight and heart weight were not different among the genotypes (Figures 1D–F).

### Loss of AT_2_R Does Not Affect Large Artery Stiffness

**Figure 2:**
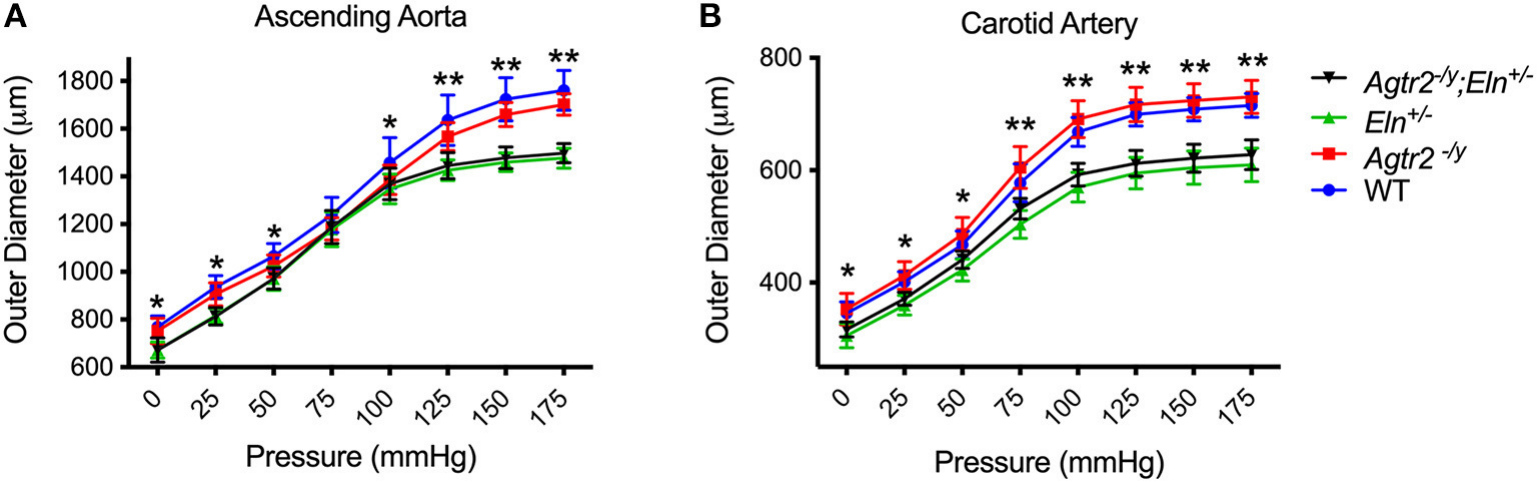
Loss of AT2R does not affect large artery stiffness. Pressure-diameter relationships of ascending aorta **(A)** and carotid arteries **(B)** from WT (*n* = 9–10), *Agtr2*^−/*Y*^ (*n* = 10–11), *Eln*^+/−^ (*n* = 6) and *Agtr2*^−/*Y*^*;Eln*^+/−^ (*n* = 15) mice. Data are presented as mean ± standard deviation. Two-way analysis of variance with Tukey's multiple comparison test was performed to compare all groups. Significant difference: *P < 0.05 and **P < 0.005 between WT or *Agtr2*^−/*Y*^ vs. *Eln*^+/−^ or *Agtr2*^−/*Y*^*;Eln*^+/−^.

One of the characteristic features of elastin insufficiency is large artery stiffness assessed by pressure-diameter curves experimentally in *Eln*^+/−^ mice and by pulse wave velocity in humans with Williams syndrome (4, 6). To determine whether the improvement in blood pressure in *Agtr2*^−/*Y*^*;Eln*^+/−^ mice was related to an improvement in large artery stiffness, we assessed ascending aorta and carotid artery mechanics in mutant and littermate control mice. As seen in Figure 2, loss of AT_2_R alone had no effect on large artery stiffness or compliance and it did not ameliorate the large artery stiffness seen in elastin insufficiency.

### Conduit and Resistance Arteriolar Structure Is Unaffected by Loss of AT_2_R

**Figure 3:**
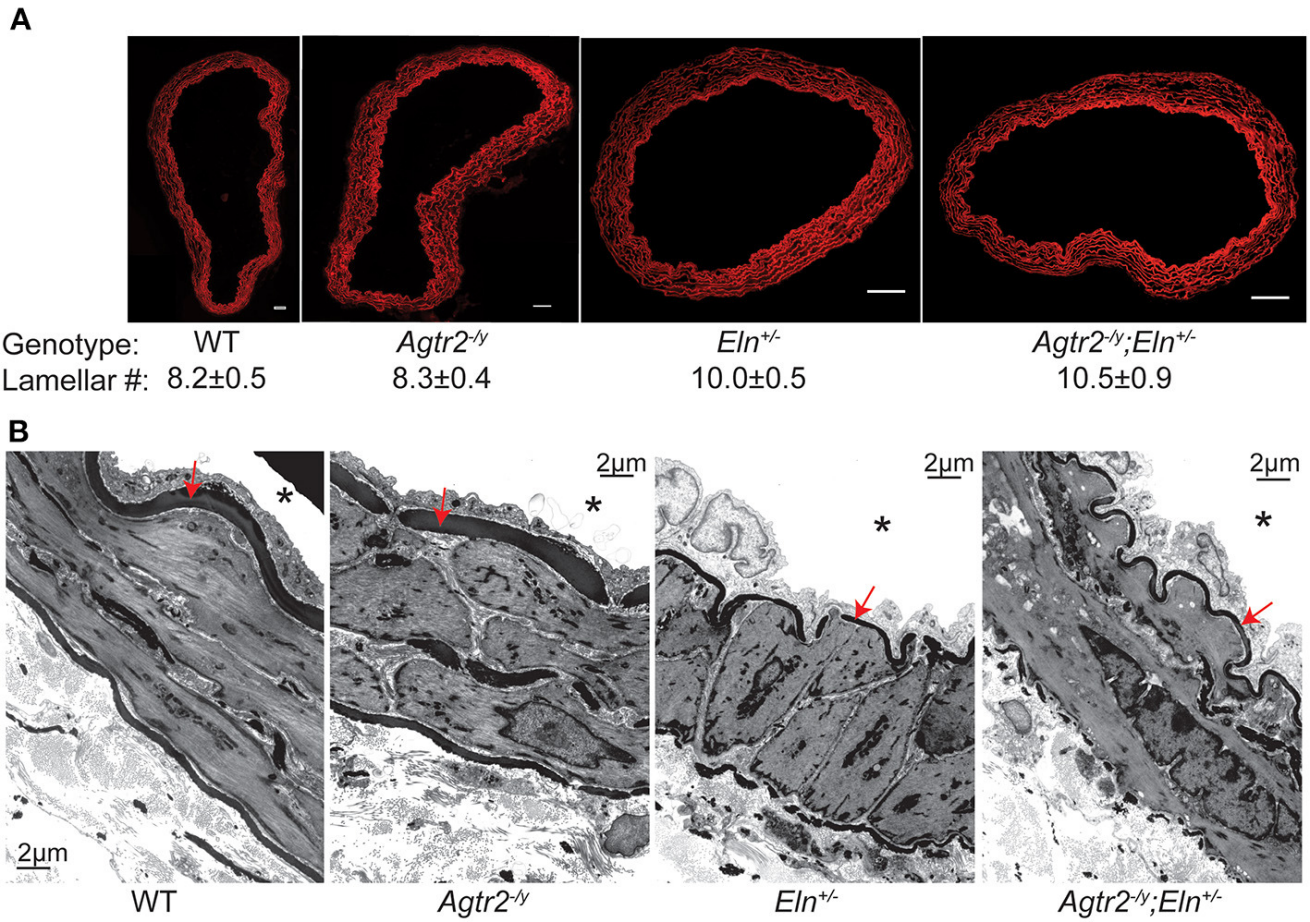
Loss of AT2R does not affect arterial structure. Representative cross sections of Alexa-633 hydrazide-stained ascending aorta from WT, *Agtr2*^−/*Y*^, *Eln*^+/−^, and *Agtr2*^−/*Y*^*;Eln*^+/−^ mice along with the respective average lamellar number ± standard error of the mean, *n* = 3–4 per group, scale bar = 50 μm **(A)**. Transmission electron micrographs of mesenteric arteries from all genotypes, *indicates vessel lumen and red arrow indicated internal elastic lamina **(B)**.

Ascending aorta of elastin insufficient mice have, on average, two additional lamellar units (7). We examined whether loss of AT_2_R has any consequences on large and small artery structure. Fluorescence microscopy using Alexa-633 hydrazide staining of ascending aorta showed that, like WT ascending aorta, *Agtr2*^−/*Y*^ ascending aorta have 8–9 lamellar units while *Eln*^+/−^ ascending aorta have 10–11. Loss of AT2R did not affect lamellar unit number in elastin insufficiency as *Agtr2*^−/*Y*^*;Eln*^+/−^ ascending aortae had 10–11 lamellar units. Representative images are shown in Figure 3A. Ultrastructural examination of mesenteric arteries by transmission electron microscopy did not identify an effect of AT_2_R on arteriolar wall structure. As previously described, the internal elastic lamina of *Eln*^+/−^ mesenteric arteries was thinner compared to WT mesenteric arteries, a finding that was not affected by loss of AT_2_R (Figure 3B).

## Discussion

Ang II, the principal effector of the renin-angiotensin system, exerts its functions in physiological and pathological states mainly through two receptors, AT_1_R and AT_2_R. In hypertension, the pathologic remodeling that occurs, including vasoconstriction, fibrosis, proliferation, and inflammation, has been attributed to ang II's actions through AT_1_R. Over the past two to three decades, a great deal of effort has focused on understanding the role of the more elusive AT_2_R. Evidence suggests that while its levels are low in the adult cardiovascular system at baseline, AT_2_R expression increases significantly in pathological conditions and it is thought to counter-balance the effects of ang II by promoting a vasodilatory, anti-fibrotic, apoptotic, and anti-inflammatory phenotype (23, 24). Often the vasodilatory effect of AT_2_R is only evident when the vasoconstrictor action of AT_1_R is blocked. At baseline, AT_2_R knock-out mice were normotensive but showed an increased pressor response to ang II infusion (20). With the availability of several non-peptide AT_2_R agonists, their use is being investigated as a potential therapeutic option in several disease conditions. In this report, based on *in vitro* data suggesting a vasocontractile role for AT_2_R in elastin insufficiency, we sought to determine whether AT_2_R contributes to elastin insufficiency-mediated hypertension *in vivo*. Using mouse models with genetic loss or insufficiency of AT_2_R and ELN, we show that, unlike its protective role in heart failure, myocardial infarction and aneurysms, in the context of elastin insufficiency loss of AT_2_R improves blood pressure making its activation a potentially detrimental therapeutic strategy in this disease state.

While initially surprising, the observation that AT_2_R may play a vasocontractile role has been made in other models of hypertension. For instance, Touyz et al. (25) showed an enhanced contractile response to ang II in mesenteric arteries from spontaneously hypertensive rats (SHR) compared to Wystar-Kyoto rats (WKY). This response was reduced by AT_2_R blockade in young but not old SHR. Similarly, coronary arteries from SHR were found to have enhanced constriction to ang II, that was attributed to the absence of counter-regulatory AT_2_R-mediated relaxation and/or a change in the AT_2_R phenotype from dilatory to contractile (26).

An interesting observation from our study is that loss of AT_2_R decreases both systolic and diastolic blood pressure, while pulse pressure, an indicator of conduit artery stiffness, remains significantly elevated in *Agtr2*^−/*y*^*;Eln*^+/−^ mice compared to WT and *Agtr2*^−/*y*^ mice. These data support the large artery pressure-diameter measurements showing that loss of AT_2_R does not affect large artery mechanics in elastin insufficiency. Rather, loss of AT_2_R likely affects peripheral vascular resistance leading to a reduction in both systolic and diastolic blood pressure.

Activation of AT_2_R by ang II has been shown to increase nitric oxide (NO) production, which activates guanylate cyclase to generate cyclic guanosine monophosphate (cGMP) leading to vasodilation (27, 28). The mechanism by which AT_2_R leads to vasoconstriction in elastin insufficiency is unclear at this time. Similar to WT, *Agtr1* is expressed at higher levels than *Agtr2* in *Eln*^+/−^ vessels, and both *Agtr1* and *Agtr2* expression levels were unchanged in aortae and reduced in mesenteric arteries of *Eln*^+/−^ mice (11), making relative changes in receptor levels an unlikely explanation for the observed blood pressure response. It is interesting to note however that, while usually thought of as monomers, G protein-coupled receptors like AT_1_R, AT_2_R and bradykinin receptor (B_2_R) have been shown to heterodimerize and adopt either an enhanced or an altered function. For instance, heterodimerization of AT_1_R and B_2_R led to increased activation of Gαq and Gαi, the two major signaling proteins activated by AT_1_R (29). This AT_1_R-B_2_R heterodimerization was shown to contribute to ang II hypersensitivity in pre-eclampsia (30). AT_2_R has been shown to dimerize with B_2_R leading to enhanced NO and cGMP (27). Since AT_2_R expression was reduced in elastin insufficient mesenteric arteries (11), it is interesting to speculate that AT_2_R-B_2_R dimer formation may be affected, or alternatively, that AT_2_R heterodimerizes with AT_1_R in elastin insufficiency, resulting in vasoconstriction rather than vasodilation; hypotheses that will be the focus of future investigation.

In summary, using a mouse model of elastin insufficiency-mediated hypertension, here we show that loss of AT_2_R improves blood pressure in this model. While the process of elastin insufficiency is distinct, with normal aging older adults develop vascular elastic fiber thinning, systolic hypertension with widened pulse pressure and large artery stiffness, all characteristics of elastin insufficient mice. Therefore, if AT_2_R agonists are to be considered for clinical use, carefully designed randomized clinical trials with special attention to patient population and endpoints will be necessary to ensure that they are not contributing to disease, particularly hypertension. AT_2_R agonists will likely be useful in a context-specific manner.

## Data Availability Statement

The raw data supporting the conclusions of this article will be made available by the authors, without undue reservation.

## Ethics Statement

The animal study was reviewed and approved by Institutional Animal Care and Use Committee of Washington University School of Medicine.

## Author Contributions

RM, BK, and CH conceived the study design. ML, RR, and CH performed experiments, generated and analyzed data. CH drafted the manuscript. All authors have read and approved the final manuscript.

## Funding

This work was supported by National Institutes of Health grants K08 HL135400 to CH and R01-HL53325 to RM. Funding for BK came from the Division of Intramural Research of the NIH, ZIA HL006210. Funds were also provided by the Ines Mandl Research Foundation to RM.

## Conflict of Interest

The authors declare that the research was conducted in the absence of any commercial or financial relationships that could be construed as a potential conflict of interest.

## Publisher's Note

All claims expressed in this article are solely those of the authors and do not necessarily represent those of their affiliated organizations, or those of the publisher, the editors and the reviewers. Any product that may be evaluated in this article, or claim that may be made by its manufacturer, is not guaranteed or endorsed by the publisher.
